# Accelerating process development through analysis of cell metabolism

**DOI:** 10.1186/1753-6561-5-S8-P81

**Published:** 2011-11-22

**Authors:** Dirk Müller, Florian Kirchner, Stefanie Mangin, Klaus Mauch

**Affiliations:** 1 Insilico Biotechnology AG, D-70563 Stuttgart, Germany

## Background

Biopharma process development accumulates growing collections of physicochemical product information and fermentation data, partly in response to initiatives like Process Analytical Technology (PAT) and Quality by Design (QbD) backed by regulatory authorities. The key goal of these initiatives is to ensure robust processes and consistent product quality based on a scientific and mechanistic understanding of the product compound itself and of the manufacturing process. Biopharma companies gather much high-quality data during fermentations including online measurements and omics data such as transcript and metabolite measurements. Typically, these data will be stored for documentation purposes only whereas data evaluation and interpretation lags behind and often is sporadic at best.

Cellular network models can tap this underused resource for predicting fermentation outcomes and for analyzing why certain fermentations failed or succeeded based on a mechanistic representation of cell physiology. In particular, network models of cell metabolism upgrade metabolomics data by enabling predictions of cell behavior from concentration time series of extracellular and – if available – intracellular metabolites. Model simulations can be used for rapid hypothesis testing, e.g. to evaluate the impact of changes in feeding on intracellular metabolism, growth, or product formation. Identifying suitable metabolic target genes for cell line engineering represents another application area of such models. Here, we illustrate this approach using the prediction of optimal media compositions for a Chinese hamster ovary (CHO) cell line employing a genome-based CHO network model as example.

## Methods

The CHO stoichiometric metabolic network was reconstructed using information from public databases as well as from primary literature and accounts for the specific amino acid composition and glycoform structure of the product molecule. In a first step, we applied the network model to a comprehensive metabolic characterization of the existing fermentation process. Rates of cellular nutrient uptake, growth, and product formation in physiologically distinct process phases were determined from concentration time series of extracellular metabolites during a fermentation run. These cell-specific rates served to compute intracellular flux distributions using the CHO network model. Comparing flux distributions for different process phases provided insight as to when and where in intracellular metabolism significant changes occur during the fermentation. This is often not obvious from inspection of concentration time series alone. Especially for fed-batch processes, multiple feed streams and volume changes due to pH control and sampling impede interpretation of raw data. If desired, further information about the usage of alternative intracellular pathways and *in vivo* reaction reversibilities can be obtained from labeling experiments combined with transient ^13^C-Metabolic Flux Analysis [1,2], which is applicable to industrial fed-batch settings.

Intracellular flux distributions also provide an ideal starting point for process optimization. Distinct optimal media compositions were computed for different fermentation phases based on the observed nutrient demand of the clone inferred from flux distributions. The chosen optimization approach combines stationary and dynamic model simulations on high-performance computing clusters. For dynamic simulations, the stoichiometric CHO network representation was transformed into a kinetic model. Model parameters were determined using evolutionary strategies and cluster computing based on the observed metabolite time series and considering thermodynamic constraints on reaction directionality. Integration of intracellular metabolite data into this workflow is easy and can further increase the predictive capabilities of the resulting model. The dynamic model also comprised a description of the fermenter including feeds and sampling. In this way, it can be predicted how changes in medium composition and feed flows impact rates of cell growth, productivity, and byproduct formation as well as intracellular metabolite profiles. Finally, media were optimized to maximize final product titer and specific productivity by varying the concentrations of glucose and individual amino acids in two continuous feed streams using evolutionary strategies on high-performance computing clusters.

## Results

**Figure 1:**
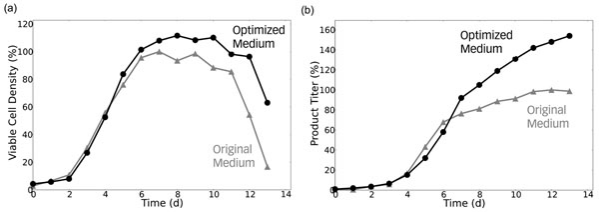
Optimized fed-batch media maintained (a) high viable cell counts and (b) resulted in a 50% increase in product titer.

The resulting optimized media were tested experimentally in a fed-batch process. The improved feeding resulted in a 50% increase of final product titer and in an increased integral of viable cells already in the first iteration (Figure 1). Simultaneously, ammonium release declined markedly. If desired, the procedure can be repeated to further optimize cellular growth and/or productivity profiles using data collected from the first evaluation fermentation as input. Considering replicate fermentations aids in assessing and improving the robustness of the predicted media compositions, but is not a prerequisite. The mechanistic model captures stoichiometric couplings between observed substrate uptake and resulting growth, product synthesis and byproduct formation. Consequently, the present approach requires much fewer fermentations runs as input for media optimization compared to standard Design of Experiment (DoE) techniques, thus saving time and resources. Presently, the prediction focuses on amino acids and carbon sources, but the extension to further compounds is technically straightforward.

## Conclusions

The combination of metabolomics data and network models not only improves our quantitative understanding of cell physiology, but can also support and accelerate multiple steps of rational process development strategies:

• Tailor media compositions to specific clones and curtail the time and experimental effort required for medium optimization compared to standard DoE techniques

• Identify highly productive and robust clones for scale-up through comprehensive metabolic characterization during selection at small scales

• Devise cell line engineering strategies for overcoming metabolic bottlenecks in cell growth and product formation by incorporating intracellular metabolite measurements and other omics data (proteins, transcripts)

• Employ metabolic network models for controlling feed additions at the production scale.

The above methodology enables biologics manufacturers to add value to data collected in PAT and QbD initiatives and to harness metabolomic data for quantitatively predicting and optimizing fermentation outcomes. This approach is not restricted to CHO cells, but is readily transferable to other cell lines and also to microbial production strains.
